# Women’s experiences of attempted suicide in the perinatal period (ASPEN-study) – a qualitative study

**DOI:** 10.1186/s12888-024-05686-3

**Published:** 2024-04-03

**Authors:** Kaat De Backer, Alexandra Pali, Fiona L. Challacombe, Rosanna Hildersley, Mary Newburn, Sergio A. Silverio, Jane Sandall, Louise M. Howard, Abigail Easter

**Affiliations:** 1 Department of Women & Children’s Health, School of Life Course and Population Sciences, Faculty of Life Sciences & Medicine, King’s College London, 10th Floor North Wing, St. Thomas’ Hospital, Westminster Bridge Road, Lambeth, London, SE1 7EH UK; 2 Department of Clinical Psychological Science, Faculty of Psychology and Neuroscience, Maastricht University, Maastricht, The Netherlands; 3 Section of Women’s Mental Health, Institute of Psychiatry, Psychology, and Neuroscience, King’s College London, Denmark Hill, 16 De Crespigny Park, London, SE5 8AF England; 4 Patient and Public Involvement and Engagement Lead for ARC South London, Maternity and Perinatal Mental health theme, Department of Women & Children’s Health, School of Life Sciences and Medicine, King’s College London, 10th Floor North Wing, St. Thomas’ Hospital, Westminster Bridge Road, Lambeth, London, SE1 7EH UK; 5 Department of Women & Children’s Health, School of Life Course & Population Sciences, Faculty of Life Sciences & Medicine, King’s College London, 6th Floor Addison House, Great Maze Pond, Southwark, London, SE1 1UK UK; 6 School of Psychology, Faculty of Health, Liverpool John Moores University, Liverpool, Merseyside, L3 3AF UK

**Keywords:** Suicide, Pregnancy, Childbirth, Perinatal, Qualitative research, Women’s mental health

## Abstract

**Background:**

Suicide is a leading cause of maternal death during pregnancy and the year after birth (the perinatal period). While maternal suicide is a relatively rare event with a prevalence of 3.84 per 100,000 live births in the UK [1], the impact of maternal suicide is profound and long-lasting. Many more women will attempt suicide during the perinatal period, with a worldwide estimated prevalence of 680 per 100,000 in pregnancy and 210 per 100,000 in the year after birth [2]. Qualitative research into perinatal suicide attempts is crucial to understand the experiences, motives and the circumstances surrounding these events, but this has largely been unexplored.

**Aim:**

Our study aimed to explore the experiences of women and birthing people who had a perinatal suicide attempt and to understand the context and contributing factors surrounding their perinatal suicide attempt.

**Methods:**

Through iterative feedback from a group of women with lived experience of perinatal mental illness and relevant stakeholders, a qualitative study design was developed. We recruited women and birthing people (*N* = 11) in the UK who self-reported as having undertaken a suicide attempt. Interviews were conducted virtually, recorded and transcribed. Using NVivo software, a critical realist approach to Thematic Analysis was followed, and themes were developed.

**Results:**

Three key themes were identified that contributed to the perinatal suicide attempt. The first theme ‘Trauma and Adversities’ captures the traumatic events and life adversities with which participants started their pregnancy journeys. The second theme, ‘Disillusionment with Motherhood’ brings together a range of sub-themes highlighting various challenges related to pregnancy, birth and motherhood resulting in a decline in women’s mental health. The third theme, ‘Entrapment and Despair’, presents a range of factors that leads to a significant deterioration of women’s mental health, marked by feelings of failure, hopelessness and losing control.

**Conclusions:**

Feelings of entrapment and despair in women who are struggling with motherhood, alongside a background of traumatic events and life adversities may indicate warning signs of a perinatal suicide. Meaningful enquiry around these factors could lead to timely detection, thus improving care and potentially prevent future maternal suicides.

**Supplementary Information:**

The online version contains supplementary material available at 10.1186/s12888-024-05686-3.

## Background

Pregnancy, childbirth, and the postnatal period are a positive and empowering experience for many women and birthing people^1^. Yet it is widely accepted that the perinatal period is also a time of significant stress, with one in four women experiencing mental health difficulties during this time [3]. Evidence on the impact of perinatal mental ill-health on the mother [4], her children [5], the wider family [6] and society [7] has grown in the last decade and worldwide, maternal suicide has been identified as a global public health issue [8]. In European countries with enhanced surveillance systems for maternal mortality maternal suicide has been identified as one of the leading causes of maternal death [9]. In the UK, the Confidential Enquiries into Maternal Deaths (MBRRACE-UK) have repeatedly highlighted similar findings, leading to the development and expansion of specialist perinatal mental health services in the UK [10]. Despite this, there has been no sign of a reduction in suicide rates [11–14]. The UK Government has therefore identified pregnant women and new mothers for the first time as a priority group in the recent Suicide Prevention Strategy [15].

While maternal suicide is a relatively rare event with a prevalence of 3.84 per 100,000 live births (95% CI 2.55–5.55) in the UK [1], many more women will *attempt* suicide during pregnancy and the year after birth. Worldwide, the pooled prevalence of perinatal suicide attempts has been estimated to be 680 per 100,000 (95% CI 0.10–4.69%) during pregnancy and 210 per 100,000 (95% CI 0.01–3.21%) during the first-year postpartum [2]. As well as distressing in their own right, perinatal suicide attempts are known to increase the risk of future fatal acts [16]. Antenatal [17] and postnatal suicide attempts [18] are also associated with increased maternal and neonatal morbidity, adverse birth outcomes, and further suicide attempts.

It is important to note that terminology in suicide research has been a contentious issue and a wide range of definitions have been used in various contexts. The US National Center for Injury and Control issued guidance on uniform definitions in the context of self-directed violence’ [19], which has informed our study definition of ‘suicide attempt’: “a non-fatal, self-directed, potentially injurious behaviour with intent to die as a result of the behaviour. A suicide attempt might not result in injury”. This definition contains three components worth highlighting, i.e. (1) suicidal ideation, (2) suicidal intent and (3) suicidal behaviour. ‘Suicidal ideation’, also known as ‘suicidality’ (i.e. thoughts of engaging in suicide-related behaviour) [19] is a known risk factor for suicide [20] but does not necessarily lead to suicidal behaviours (e.g., behaviour that is self-directed and deliberately results in injury or the potential for injury to oneself, with implicit or explicit evidence of suicidal intent’) [19]. ‘Suicide attempt’ must also be distinguished from ‘near-fatal deliberate self-harm’, which was defined by Douglas et al (2004) as ‘an act of self-harm using a method that would usually lead to death, or self-injury to a “vital” body area, or self-poisoning that requires admission to an intensive care unit or is judged to be potentially lethal [21]’. This definition does not contain an element of ‘suicidal intent’, ie. explicit or implicit evidence that at the time of injury the individual intended to kill self or wished to die, and that the individual understood the probable consequences of his or her actions [19].

To date, perinatal suicide research has predominately been based on case note reviews [1], retrospective cohort studies [22], or qualitative studies focussing on suicidal ideation [23]. Research into suicide attempts in the perinatal period is therefore acutely needed, to gain a better understanding of the circumstances surrounding maternal suicide, the support available to perinatal women and how future deaths can be avoided. To our knowledge, no studies in the UK have used qualitative methods to explore the experiences of women who undertook a suicide attempt in pregnancy or during the postnatal period, yet survived. A better understanding of these events could help refine support and early interventions for women and birthing people at risk.

## Methods

### Aim of the study

The aim of this study was to explore the experiences of women and birthing people who had undertaken one or more suicide attempts during the perinatal period.

### Study design

The ASPEN-study (Attempted Suicide during the PEriNatal period) utilised a qualitative design, using semi-structured interviews, to allow for an in-depth understanding of the contextual factors of perinatal suicide attempts, and to demystify the taboos and misunderstanding that are enshrouding this phenomenon [24]. Qualitative methods are particularly helpful to study sensitive topics [25] and can facilitate a deeper understanding of suicide attempts, beyond merely explaining [26]. We adopted a critical realist ontology, meaning participants’ accounts were seen as ‘truths’, even when their reported recall might have been impacted by serious mental illness and/or distress at the time of events [27]. We also adopted an objectivist epistemological stance meaning our belief system of how we acquire knowledge is one of reality existing and not being constructed, thus enabling an approach to participants’ narratives with no preconceived notions of how the participants may experience the phenomenon of interest [28]. Drawing on our epistemological and ontological positions, a critical realist approach to Thematic Analysis was best aligned with our philosophical underpinnings. Critical realist TA is an alternative approach to Thematic Analysis, that differs from codebook TA with its positivistic assumptions [29], or reflexive TA that is grounded in philosophical constructivism [30, 31]. Critical realist TA is an explanatory approach that aims to produce causal knowledge through qualitative research on phenomena in the world around us [32, 33]. We wanted to go beyond merely ‘exploring’ the phenomenon of perinatal suicide attempt, but aimed to understand what women had experienced during this time, such as any significant life course events they identified as relevant to their perinatal suicide attempt, the specific circumstances in the lead-up to the suicide attempt, their views of motherhood and how this impacted their mental health and any key elements or milestones that made a substantial difference on their journey to recovery. As such, this approach informed our development and structure of the interview schedule and analysis of the data to ensure that this was captured.

### Participants and recruitment

The study was advertised through social media and third sector organisations in the field of perinatal mental health and suicide prevention (see Acknowledgements). Interested participants were included if they: (1) were 18 years of age or older; (2) had one or more suicide attempts during the perinatal period (i.e. from pregnancy up to the first year after giving birth), including when the attempt was prevented by self, a loved one or a member of the public; (3) and this happened less than 10 years ago; (4) were residing in the UK; and (5) were not receiving inpatient psychiatric care or experiencing an acute episode of a psychiatric disorder at the time of recruitment. The latter exclusion criterium was adopted in line with our safety protocol, to prevent delays in recovery by addressing such a difficult event outside a therapeutic environment. We used both convenience sampling and purposive sampling techniques: we interviewed anyone who responded to our recruitment materials, met the inclusion criteria and wanted to participate in the study after reading the participant information sheet (convenience sampling). Simultaneously, we also made concerted efforts through intense collaboration with community leaders and third sector organisations to recruit a diverse sample of women and birthing people from different ethnic, cultural, socio-economic and religious backgrounds (purposive sampling). A total of twelve women and birthing people contacted the research team with an interest in the study. Eligibility for the study was explored in a sensitive way, against the overall inclusion criteria and the three components of the study’s definition of ‘suicide attempt’ (suicidal ideation, intent and behaviour). Where in doubt, eligibility was discussed with the wider supervision team. In total, eleven interviews were conducted. A twelfth interested participant did not attend the (online) interview and did not respond to any follow-up emails. Recruitment was finalised when no new themes were being generated from data analysis of the last two interviews [34]. Participants received reimbursement of £50 for their time to complete the interview and a short demographic survey.

### Data collection and analysis

Semi-structured interviews lasted between 38 and 115 min (*MTime* = 65 min) and were conducted via video-conference software (Microsoft Teams) by one researcher (KDB) between October 2022 and April 2023. Interviews were audio-recorded, transcribed and de-identified by a professional transcription company. Field notes were taken during the interview. Transcriptions were checked for accuracy by two researchers (KDB, AP). The interview schedule, which was co-designed with a panel of women with lived experience of perinatal mental illness, aimed to explore experiences of mental health difficulties prior to and during the perinatal period, the circumstances in the lead-up to the suicide attempt, and those following the suicide attempt. The interview schedule was used flexibly and did not prevent participants from sharing their story in the order they preferred, but instead, was used as an aid to prompt where required. Interview data was so rich that a secondary analysis focusing on social support prior and after women’s suicide attempts was undertaken, to be published separately.

**Fig. 1:**
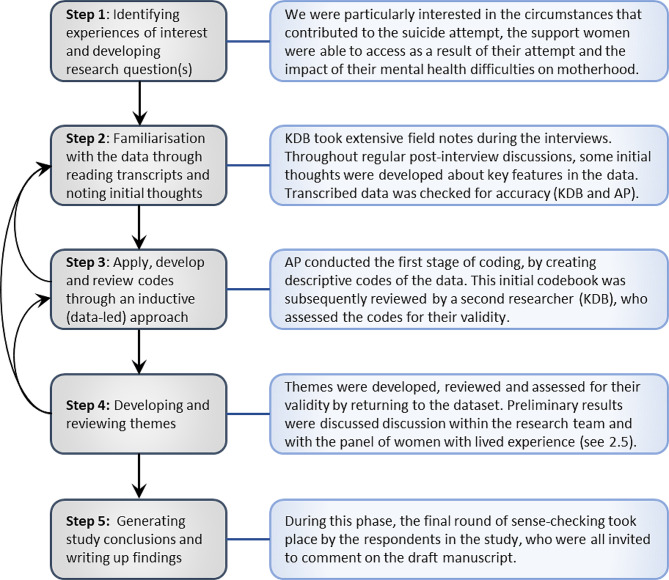
Display of critical realist approach to thematic analysis

Thematic Analysis (TA) [30, 31, 33] of the interview data was conducted using NVivo software while adopting a critical realist approach to Thematic Analysis [30, 31, 33]. The process of data analysis is rarely a linear event, and guided by Fryer’s previous work on critical realist TA [33], our approach to data analysis is presented in Fig. 1 and can best be described as follows:

### Public and patient involvement and engagement (PPIE)

An established advisory panel of women with lived experience of perinatal mental illness was consulted during different phases of the study with additional feedback sought from key stakeholders in the field of perinatal mental illness (see Acknowledgements). The process of PPIE during the study design and data collection phase of this study has been documented elsewhere [35]. A draft manuscript was shared with research participants to sense-check findings and comment on the manuscript. Participants were also given the opportunity to select a pseudonym of their choice. A total of 8 participants reviewed the draft manuscript and their feedback was incorporated in the final version of this paper.

### The study team and reflexivity

The research team are a multidisciplinary team of researchers and clinical academics, with backgrounds in psychology (FLC, AE, RH, SAS, AP), psychiatry (LMH), and midwifery (KDB, JS), and several had clinical experience of supporting women who attempted suicide during the perinatal period (KDB, FLC, LMH). Within the research team, there was a balance between those who were parents and those who did not have children and researchers were at different stages of their life, spanning nearly three generations. The phenomenon of suicidality in the perinatal period was familiar to most of the research team, through extensive clinical experience and/or previous research in the field of perinatal mental health. Our positionality is therefore best described as ‘hybrid’, concordant with our critical realist ontology, as we aimed to align our existing knowledge and understanding (i.e. being embedded in the data) with the uniqueness and unfamiliarity of each individual story that was shared with us as a ‘truth’ (i.e. being an objective onlooker), in order to analyse the data in a coherent and sensitive matter [36]. Data were collected by one researcher (KDB) who was trained in advanced qualitative research techniques as well as having clinical experience as a perinatal mental health midwife. Analysis was conducted by the same researcher and a MSc Student with a background in clinical psychology (AP). Regular team meetings were held throughout the data collection and analysis phase to discuss and sense-check the developing themes and sub-themes.

### Participant safety and researcher wellbeing

The safety and emotional wellbeing of all participants was key throughout the study. Thus, we adopted key elements of trauma-informed care into our study design [37]. A robust safety protocol, with clear pathways for escalation if required, was developed with the input of the PPIE advisory panel [38]. The study team undertook bespoke training in trauma-informed interviewing and the interview schedule was developed with this in mind. A safety check prior and after the interview was carried out by the same researcher (KDB), either via email or by phone and all participants were offered a confidential de-brief session with an independent clinical psychologist. The psychological safety of the researchers was also considered [25] and supported by access to regular reflective supervision sessions provided by a clinical psychologist and regular debrief sessions with supervisors to process any difficult emotions arising from conducting the interviews [39]. We were acutely aware of the potentially triggering content of the audio files and raised this with the transcription company [40]. When sending audio recordings for transcription, a summary of triggering content was provided to ensure the transcription would be appropriately allocated.

## Results

The majority of our sample (*N* = 11) were White British women (*n* = 10), with one woman from a mixed ethnic background. Participants were predominantly married (*n* = 8) and had higher education qualifications (*n* = 7). All but one participant had received a mental health diagnosis by a doctor or other healthcare professional in the past although the demographic survey did not allow to ascertain when this diagnosis had been given. More than half of the participants in our sample were given multiple diagnoses, indicating a high level of complexity in mental health presentation. In most cases, pregnancies had been planned (*n* = 9). All but two women were multiparous, with half of the sample having two children (*n* = 6), and three participants having three or more children. Two women were first-time mothers at the time of the attempt. Four women undertook their suicide attempt during pregnancy, with a fifth woman being pregnant whilst her older child was still under the age of one. The remaining six women undertook a suicide attempt within the year after giving birth. Four participants had a stay in an inpatient psychiatric Mother and Baby Unit (MBU), and for three of them the admission was preceded by their suicide attempt. For one participant, the admission in the Mother and Baby Unit was subsequently followed by an admission in a general psychiatric hospital, where she undertook the actual suicide attempt. A full table of demographic and clinical information can be found in Table 1.

**Table 1:** Participants’ demographic and clinical characteristics

Demographic and clinical characteristics	Total *N* = 11	
**Age**		
25–34	*n* = 3	
35–42:	*n* = 6	
Over 42	*n* = 2	
**Ethnicity**		
White (English)	*n* = 10	
Mixed or Multiple Ethnic groups	*n* = 1	
**Marital status**		
Single	*n* = 1	
In a relationship -currently living together	*n* = 1	
Married	*n* = 8	
Divorced or in legally dissolved same-sex civil partnership	*n* = 1	
**Parity at index pregnancy preceding the suicide attempt**		
Primiparous	*n* = 2	
Multiparous	*n* = 9	
**Number of children**		
One child	*n* = 2	
Two children	*n* = 6	
Three children	*n* = 1	
Four children	*n* = 1	
Five or more	*n* = 1	
**Highest level of education**		
No educational qualifications	*n* = 1	
GCSE equivalent (in secondary education up to 16y)	*n* = 2	
A levels or equivalent (in secondary education up to 18y)	*n* = 1	
Undergraduate degree or equivalent	*n* = 2	
Postgraduate degree or equivalent	*n* = 5	
**Received a formal mental health diagnosis**		
No	*n* = 1	
Yes, single diagnosis	*n* = 3	
Yes, multiple diagnoses	*n* = 7	
**Mental health diagnoses**,		
Psychotic mental health disorders	*n* = 3	
Non-psychotic mental health disorders	*n* = 7	
Missing	*n* = 1	
**Planned pregnancy**		
Planned	*n* = 9	
Unplanned	*n* = 2	
**Timing of suicide attempt**		
During pregnancy	*n* = 4	
During the postnatal period (within one year after birth)	*n* = 6	
During the postnatal period, already pregnant with subsequent child	*n* = 1	
**Classification of suicide attempt (ICD-11 classification of intentional self-harm)**	**Actual** ^*^	**Prevented** ^**^
Fall or jump	*n* = 1	*n* = 1
Transport injury event	-	*n* = 1
Exposure to or harmful effects of substances	*n* = 3	*n* = 1
Threat to breathing	*n* = 1	*n* = 2
Self-harm by sharp object	*n* = 1	**-**
**Highest level of care after the suicide attempt** ^**+**^		
Admission to an inpatient psychiatric unit, incl. Mother and Baby Unit	*n* = 4	
Community mental health Service, incl. Community Perinatal Mental Health Service	*n* = 5	
Accidents and Emergency (A&E) Department	*n* = 1	
Primary Care (General Physician)	*n* = 1	
**Admission to general hospital as a result of suicide attempt, requiring treatment**		
Yes	*n* = 3	
No	*n* = 8	

^*^We refer to an ‘actual’ suicide attempt when a suicide plan was carried out yet was not fatal

^**^ We refer to a ‘prevented’ suicide attempt when respondents were stopped before or in the middle of the attempt, either by their own actions, or those of a third party

+ ‘Highest level of care’ refers to the level of care received as a direct result of the suicide attempt and the episode of acute mental health crisis surrounding the attempt

**Fig. 2:**
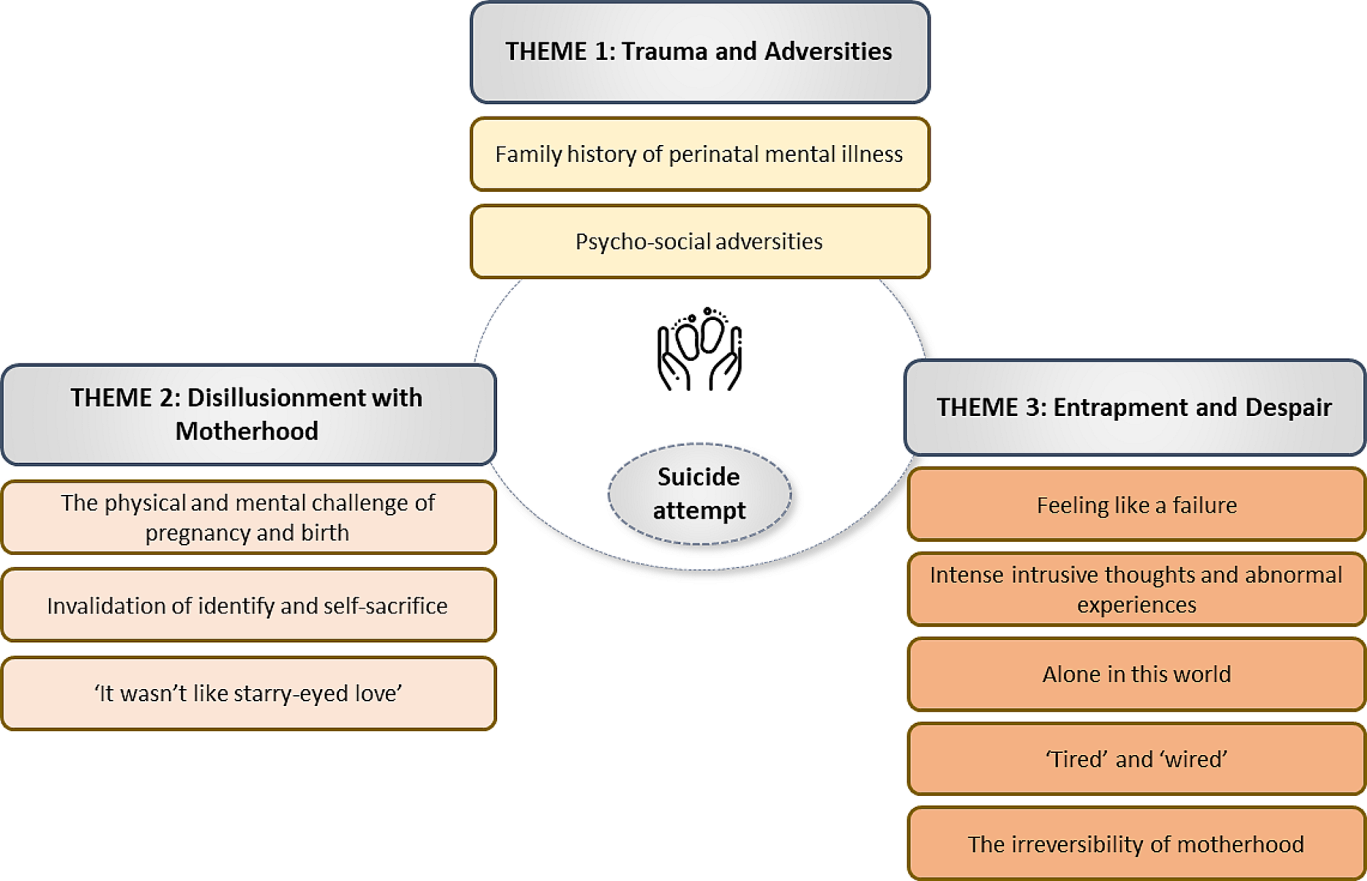
Display of themes and sub-themes

Qualitative analysis resulted in the identification of three key themes that played a significant role in the deterioration of women’s mental health during the perinatal period, ultimately culminating into a suicide attempt. Saturation for all themes and sub-themes was achieved after nine interviews when no new themes or subthemes were generated. Data from the remaining two interviews confirmed our analysis and provided additional depth and detail [34]. The three overarching themes are presented in Fig. 2: *Theme 1 ‘Trauma and Adversities’*, consisting of family history of perinatal mental illness and psycho-social adversities, including grief and trauma; *Theme 2 ‘Disillusionment with Motherhood’*, marked by a variety of challenges that arose during pregnancy or the postnatal period; and *Theme 3 ‘Entrapment and Despair’*, where multiple stressors piled up with no respite or support available, leading to a severe deterioration of mental ill-health, and ultimately, the suicide attempt.

Qualitative data is presented below, with the most representative quotations in text and an additional table of supplementary of quotations included in Supplementary Material 1.

### Theme 1: trauma and adversities

All respondents in our sample started their pregnancy journeys with a range of vulnerabilities, such as previous mental health difficulties, loss, trauma, or social risk factors including domestic abuse and substance misuse. Nevertheless, participants were not always aware of the profound impact these would have on their mental health later in pregnancy and in the postnatal period. Subthemes contributing to this were:

#### Psycho-social adversities

Many women had experienced mental health difficulties at some point in their life, and most were fully aware of their potentially devastating impact. Some had experienced poor mental health during adolescence and young adulthood and anticipated mental health problems during the perinatal period.

*“I’ve had some terrible things happen in my life about failed marriage and fertility problems. Big, big things that I’ve sort of managed with a strength of mine that I perhaps didn’t have in my late teens or early 20s to overcome. So I guess it was always on my radar knowing the stats around you are more likely to have perinatal mental health problems if you’ve had bouts of depression in the past.” – Rosy*.

In contrast, others had dealt with traumatic experiences in their life, but could not see how this would be relevant to their mental health during pregnancy and the postnatal period. They started their pregnancy unaware of any potential risks to their mental health.

*“I lost my brother when he was 18. […] And I didn’t get a lot of time off work, I was kind of straight back into work. I’m a [professional role in mental health], so I was working in acute psychiatry. Back to work, dealing with other people’s trauma and I don’t think I really dealt with my own particularly well. And it was kind of I think eight months later I had an episode of depression, just very low mood, apathy, poor motivation, poor concentration, was treated briefly with antidepressants and then just kind of did okay after that. So there had been nothing.” – Simone*.

Previous trauma was reported by almost all respondents, whether it being through a bereavement, or traumatic life experiences, such as miscarriage and infertility, domestic abuse, fractured relationships, or suicide of a loved one. Two women reported having experienced domestic abuse. One of them reported the abuse, which she described as a ‘punishment’, only started after informing her partner of the pregnancy.

*“It was a punishment actually that I dared to be pregnant even though he knew I wasn’t on any contraception or anything. And it really shocked me because he had never ever been like that before.” – Lauren*.

For the other respondent, the domestic abuse had been long-lasting and led her to seek coping strategies to deal with the trauma and pain. Being in an abusive relationship created the worst possible start for pregnancy, with no support available.

*“Well, it was my first pregnancy. I was 24 so I still hadn’t grown up properly, and I was in a really bad domestic violence relationship so there was a lot going on around that. I was getting no support [for] my pregnancy. I was also using as well which I regret profoundly, but I was drinking, like I drank occasionally because of my mental health, and my mental health was just all over the place; I was really, really unwell.” – Selina*.

For some, their previous mental health difficulties were related to an earlier pregnancy or birth experience:

*“I had huge amounts of birth trauma from my first, which I had a debrief for from the hospital, which was incredibly unhelpful. And it ended in emergency caesarean [section], after nine days of labour, and being in hospital, as a very naïve 19-year-old, having her first baby; looking back on it, feeling quite coerced by doctors, but not realising at the time that that’s what was happening. And that has impacted me for the rest of my life.” – Sam*.

The severity of previous perinatal mental health problems was varied, with one woman having experienced postpartum psychosis after the birth of her first child. Going into the second pregnancy, the risk of relapse was hanging over her like a dark cloud:

*“I remember sort of going to the 12-week scan with [second pregnancy] and getting the picture and thinking like shit, it’s really real now and it could all happen again. So I was really scared about that. Because the reccurrence rates are quite high for psychosis, so it’s quite likely that I was going to become unwell. So I was worried, yes, I was really concerned.” – Marie*.

This feeling of worry was also reported by women with mild to moderate mental health difficulties and was compounded by a fear of being dismissed and not being able to access support if they would require it.

*“I think there was something about the anxiousness of doing it all again, because I think I had some prenatal depression with my first, that wasn’t picked up, and then postnatal anxiety through the roof, that was also never picked up, and was told that was normal.” – Sam*.

#### Family history of perinatal mental illness

Several respondents had a family history of perinatal mental illness and were vigilant that they might experience something similar. To mitigate this risk, they actively sought perinatal mental health support at the earliest opportunity.

*“My mum had severe perinatal mental illness, she was hospitalised after my older brother for a year without him […]. At the time they didn’t really have Mother and Baby Units. Then I came [a few] years later and she was hospitalised again but with me for six months, and she passed away […] So my dad said she was saying the same things as each time she’d been sectioned; she would present with very religious ideation and stuff like this, so it was exactly the same stuff, and she died by suicide. So because of that collective history, when we were trying to get pregnant we thought “We need to let someone know we’re trying to get pregnant,” and so I was referred then to a Perinatal Psychiatrist before we got pregnant” - Sarah*.

For others, this family history was not something which was spoken about prior to their own experiences of perinatal mental illness. One respondent mentioned she had never been aware of her mother’s history of postnatal depression until she herself started to experience postnatal depression.

*“I didn’t know that my mum had postnatal depression. That’s not anything that she’d shared until… I knew that my brother cried a lot and I think he had a cows’ milk protein intolerance, but I didn’t know that my mum…” – Rosy*.

### Theme 2: disillusionment with motherhood

While previous mental health challenges or trauma were present in the background, all women were profoundly disillusioned with motherhood which contributed to a deterioration in their mental health. This theme of ‘Disillusionment with Motherhood’ captures three sub-themes that reflect a discrepancy between what women thought or hoped motherhood would be like, and the crushing reality they found themselves in. Together, these sub-themes compounded each other and became a catalyst for worsening mental health. The following sub-themes address the various areas of disillusionment that women in our sample reported: in their bodies, in their identity and in the bond with their baby.

#### The physical and mental struggle of pregnancy and birth

All participants held hopes and expectations of what their pregnancy, birth or the postnatal period would be like. For some first-time mothers, it soon became clear that the societal rosy-hued image of pregnancy was very far removed from their own experience of pregnancy. As they came to grips with how pregnancy was unfolding, the harsh contrast between expectation and reality was so high that many struggled to adjust to this:

*“There’s all this thing about pregnancy you’re supposed to be glowing and it’s all marvellous and you’ve got these wonderful hormones, but I was just beached on the sofa feeling hot and sweaty thinking when is this baby going to come out, when’s it going to come out?” – Simone*.

For those who had been pregnant before, the reality of another pregnancy, knowing full well what was in store, started to dawn on them:

*“I don’t know, it hit me like a ton of bricks. Like oh shit, I’m doing this again. I’m pregnant again.” – Liv*.

In addition to these psychological adjustments to reality, respondents mentioned how the physical toll of pregnancy and childbirth played a significant role in the deterioration of their mental health. This close correlation between physical issues and mental health decline was abundantly clear across the sample.

*“I was horribly, horribly sick [hyperemesis]; that got worse each pregnancy. I don’t know if that’s normal; I’d heard it is. But horribly sick, which makes you absolutely miserable anyway.” – Sam*.

*“I just sort of couldn’t wait for it [the pregnancy] to end. Yes, I just wanted to give birth. So when they said that they were going to induce me at 40 weeks I thought thank goodness, because my sickness started again quite late on. Again, I don’t know if it was because of the pre-eclampsia. But yes, I was just very ready, very ready to have little one.” – Hannah*.

In the most extreme cases, pregnancy was not viewed as something to be enjoyed, but something that left women feeling repulsive.

*“So since the pregnancy, just my life fell apart really, I was unemployed, and I just felt the whole way through not just sick and ill, absolutely physically repulsive, like I just felt like an absolute filthy animal. I can’t describe the disgust I felt for myself and the bigger my bump grew, the more disgusting I felt. And I don’t know, it’s just everything was awful, every day was awful.” – Lauren*.

For other women physical injuries as a result of childbirth left them unable to function and to enjoy the things they were looking forward to as a new mother.

*“I had some tearing and I’d had an episiotomy and they hadn’t healed, so my episiotomy had opened up and there were lots of A&E [Accidents and Emergency] visits and an operation eventually, but I think that really didn’t help my mental health because obviously if you’re in pain all the time then, it just drags you down, doesn’t it? So I wasn’t able to do my normal stuff, I wasn’t able to just carry on with life because I was in pain, I couldn’t sit and I felt like I couldn’t do mummy things.” – Mel*.

Apart from the physical repercussions of pregnancy and childbirth, it was the trauma of giving birth and its psychological sequalae which triggered a marked deterioration in the mental health of several women in our sample.

*“It was just sort of like you couldn’t expect it to happen, it was like a poor pregnancy and sort of felt like, you know, the birth went wrong as well.” – Hannah*.

*“I had a premature baby. And I went on, I don’t know, like trauma response. Like totally numb. I suppose the adrenalin, the shock, everything…” – Liv*.

#### Invalidation of identity and self-sacrifice

Almost all respondents encountered negative experiences with healthcare professionals at some point during pregnancy or the postnatal period and felt invalidated and dismissed by these. Women reported they were not seen as a person, with a complete identity, but reduced to a vessel for their baby, with little consideration given to their own feelings. This led to a profound loss of identity, exacerbating feelings of being invisible, inadequate and unimportant.

*“It was never about me. And I know it’s not all about me, but when I’m wanting to commit suicide, it is very much about me and not one person asked me if I was alright, they were more concerned if the baby was alright, which I was as well, but they just completely bypassed that there was any reason I would do it.“ – Selina*.

There seemed to be a lack of professional inquisitiveness to understand why a mother(-to-be) would consider suicide. Instead, all attention was directed towards the well-being of the baby, leading to multiple missed opportunities for timely care and support. In some cases, women reached out but their calls for help were simply ignored while their mood was rapidly deteriorating. These experiences would have devastating consequences on their further help-seeking behaviour.

*“What really killed me, what was like the punch in the face that I needed was when I had my midwife appointment at, I don’t know, eight, ten weeks, something like that, and I told her ‘you know what, I’m not feeling right. There’s something bubbling inside me that is not alright, is not correct. I feel more anxious than normal, I can’t sleep, it’s all very weird’. And she just said ‘okay, I’m going to pin that down here to talk about in your next appointment. But we’re not going to do anything right now’. I never saw her again, by the way.” – Liv*.

Invalidating encounters like the one described above would have a profound impact on how women viewed healthcare professionals as a source of support and whether they would reach out to them and share the extent of their mental health problems.

*“I just felt like nobody was listening at all, just not heard one bit.” – Anna*.

For some, the invalidating experience would almost become a motivator to succeed in their suicide plans, as they felt the severity of their mental health problems was brushed under the carpet. One participant sought help after a first suicide attempt through a medication overdose and shared the following:

*“So then I think a few more weeks went by and I went back to the doctor’s. I said to the doctor, ’I want to kill myself’. My medication and stuff, I was honest with him, I said the medications and stuff that he was put… I think he tried me on Zopiclone as well with not sleeping and he said, ‘Well if you wanted to kill yourself, you would have done it by now’. I was just… I sort of felt then I’ve got something to prove.” – Hannah*.

The loss of identity made respondents feel invisible to healthcare professionals and went hand in hand with exhausting themselves to be the best possible mother for their baby. Women described feelings of total self-sacrifice to meet this perceived standard of ‘the perfect mother’.

*“I think I sort of went into supermum mode when I came home, like I had something to prove, and again, it’s that background of failure. I think I’m quite hard on myself anyway and I’m quite… If something goes wrong, I’m probably harder on myself in my head than somebody else would be and I maybe got a bit of a perfectionist trait, so I really didn’t want to rely on anybody, I didn’t ask for help with anything regarding my little boy, and I had a really, really strong bond with him which was really positive, but I think I was sort of going like overkill with not asking for help.” – Hannah*.

However, as women started to experience the hard reality of caring for a newborn, they felt unable to meet this impossible standard. The perceived pressure to achieve (unrealistic) goals as well as their feelings of failure to do so started to take a significant strain on their mental health.

*“No one had ever told me that before. No one had ever said that you don’t just have to drop everything and run to your child. Because I thought that that was what a secure bond was; and obviously now I’ve learnt about attachment theory and things. I thought that, for her to be securely bonded with me, I had to give every last drop of myself to be her mum.” – Sam*.

#### ‘It wasn’t like starry-eyed love’

Closely linked with the previous sub-theme, was the realisation for many women that they did not feel an instant rush of love for their baby. Several women reported feeling unsettled and flawed as a mother when they felt distant and detached from their baby. Women tried their very best to ‘act as a mother’ and do whatever their baby required, but this did not mean they also ‘felt like a mother’.

*“So, at the beginning it was very strange. It, because like I said, I was determined to do anything in my power to get that baby out of NICU [Neonatal Intensive Care Unit]. Like whatever it takes, whatever the cost. So it never felt like oh, it’s my baby. I would have jumped in front of a train for him but it was not like a starry-eyed love. And that kept going.” – Liv*.

*“In terms of motherhood, yes, I don’t know whether I just felt I was failing at it or… [pause] I don’t know, I felt very not connected to the baby. I had felt very, very bonded and very connected, and then I wasn’t at all.” – Sarah*.

Sadly, for some, this lack of bonding with their baby persisted for a long time, with enduring consequences on their mental health and family happiness, leading to feelings of guilt and shame with which they still are coming to terms with.

*“I had no attachment to him probably for about five years, nothing at all, just this ongoing sense of regret and I remember thinking daily I’ve made such a massive mistake in my life and almost this like realisation you are never going to get back what you had before, so just this real hopelessness actually at life.” – Lauren*.

*“I just couldn’t, I couldn’t bond with, I couldn’t. Even still now I love her to pieces but we’re not like mother and daughter, we’re not.” – Anna*.

### Theme 3: entrapment and despair

In the final phase leading up to the suicide attempt, women experienced an accumulation of stressors, unleashing an overwhelming feeling of hopelessness and entrapment, with seemingly no way out of the situation they found themselves in. The sub-themes identified under this theme of ‘Entrapment and Despair’ left women no breathing space or respite. A perfect storm was brewing, for which women only started to see one way out, and that was by taking their own life.

#### Feeling like a failure

All respondents expressed a pervasive feeling of utter failure, intersecting their different identifies as a woman, mother and partner. Their perceived inability to meet expectations, whether this related to giving birth, feeding their baby, or functioning as a mother and partner stood in sharp contrast with how they viewed other mothers, who seemed to be effortlessly successful in doing so.

*“You sort of just blame yourself. So I can just remember looking at him when he was asleep thinking like, ’Oh you’ve failed, I can’t do this, I’ve already failed at being a mum, but I can’t do this’, and I can just remember just thinking that, looking at him. So I think even though I know it wasn’t my fault, you really felt like a failure and I felt like it was me, like there was something wrong with me, because a lot of women around me, like even family, they never really had experiences like that, they would have like a good pregnancy, like a vaginal birth, a normal birth, so I really felt like I had failed and I really blamed myself for that.” – Hannah*.

This feeling of being a total failure created a sense of dread, leaving them fearful every day that their inability and incompetence as a mother would be further exposed.

*“I remember seeing the light coming in through the curtains in the morning and just thinking “Oh my god, no, I can’t, I can’t do another day,” like my heart would go, and it was that dread, that whole dread would come over me and I’d think “I can’t do another day today, I just can’t do it. I can’t do it.” It was like a… Yes, it was really hard. I just felt like I don’t know, it felt like I just wasn’t good enough for her, I wasn’t good enough. […] It just felt like I wasn’t good enough to be her mum.” – Mel*.

This overwhelming sense of incompetence erased feelings of love, enjoyment or hope and instilled a feeling that their baby and loved ones would be better off without them.

*“So it just escalated. This what was going on in my head about, you know, me not being good enough, a failure, just escalated even more, that now I was thinking they are going to take him away, everyone will know how rubbish I am. So it was later that week where I still wasn’t sleeping and I just thought, do you know what, the both of them would be better off without me, because I’ve just failed, I’m just a failure. They will be better off without me.” – Simone*.

*“…That just made me feel so, so low that I think that spiral of internalised feelings and negativity compounded with this sort of isolation and lack of sleep just led me to think they’d be better off without me around, they’d have a parent maybe or a family that would be able to meet their needs.” – Rosy*.

#### Intense intrusive thoughts and abnormal experiences

More than half of the women in our sample reported intrusive ideas or unsettling experiences in the period preceding their suicide attempt. For many, this came as a total surprise as they were unaware this could happen and they felt unable to express the extent of their intrusive thoughts to anyone.

*“I remember getting up and going to the bathroom to brush my teeth and then started hearing voices. So this voice, I didn’t recognise it, was just chanting, ‘stinky [name of baby], stinky [name of baby]’, which is my baby’s name and I was like why’s that happening? I don’t understand. Where’s that coming from? And then later that day I remember looking at my husband and thinking you’re the father of this baby, but I’m not its mother. It was a really odd thought, because I was like I know I’ve been pregnant and I know I’ve just been through all that labour, but I look at this baby and it’s not mine, but I know you are the dad. It was really odd.”- Simone*.

*“They [the intrusive thoughts] were really, really scary. And totally uncontrollable as well. They were so vivid and they used to make me feel really upset because they happened quite early on, probably when she was only a few weeks old and I remembering googling them and reading loads of things about it didn’t mean that you were not coping, it didn’t meant that you were going to hurt your baby, it didn’t mean that you were depressed, but I think maybe I should have perhaps seen that as a bit of a sign that I needed to get some help because it was weeks and weeks later that I finally did. But yes, they did upset me and I only told my mum, I didn’t tell anybody else because I just felt as though are people going to think that I’m going to hurt her? Am I going to hurt her if I talk about it more? Yes, they were really scary.” – Rosy*.

For some, these ideas were extremely horrific and a symptom of their psychotic illness at the time. Unfortunately, this was left undiagnosed and untreated, leaving them totally desperate and isolated while these unsettling thoughts became their lived reality.

*“[…] I started to think ‘oh I’ve committed all these awful crimes in my life’ and I was kind of struggling to process what they were and I was thinking have I killed people and maybe buried them and I don’t know where they are or have I kind of done a big theft or something but not been able to quite work out where I’d stolen the money from. But I was kind of panicking that I’d either buried these bodies or hidden this money and I couldn’t remember where they were, so I was panicking someone else is going to find them and then I’m going to be put in prison. So I had this kind of I want to die because I’m scared I’m going to go to prison because I’ve done all these awful things. And I just felt absolutely desperate.” – Lauren*.

#### Alone in this world

While these distressing experiences of failure and intrusive thoughts invaded women’s mindset, women felt profoundly alone and isolated. Social isolation was reported as a catalyst for their suicide attempt by every woman in the sample. For some, it was a continuation of the situation they had already been in, but during this stage everything felt more desperate, more alone.

*“I think by that point I wasn’t talking to anybody at all, not family, certainly not the kids’ dad. The kids’ dad… […] I just totally blocked his number and I wasn’t seeing anybody else. And actually, in some ways, I don’t think anybody wanted to see me because they were just like, “Why have you had another kid?” So the only people that I saw were my own kids, maybe the odd school teacher at pickup but that was it. No one from work. No friends really.” – Lauren*.

For others, it was the absence of their partner, who had to return to work after paternity leave, that served as a lever for an acute deterioration of their mental health.

*“Everything was fine until about three weeks after the birth and we were back at home, and my husband went back to work; it was him going back to work and I just, yes, fell apart.” – Sarah*.

Some respondents had their baby during one of the COVID-19 pandemic lockdowns, when social restrictions meant they were unable to meet with friends or family or seek peer support from other mothers. Instead, they felt cooped up inside their house, alone and isolated, with their suicidal thoughts.

*“Completely isolated. Not being able to, like I could have been going to, I don’t know, prenatal yoga. Or breastfeeding groups or toddler groups. Anything else that would take me out of that loop. So I think obviously that made it a lot worse. I don’t think that it would have been… – I don’t know.” – Liv*.

*“So she was three or four months old when Covid hit and it was the whole lockdown and yes, everything just got ten times worse because I couldn’t do anything then; I couldn’t go and talk to my mum, I couldn’t go out, I couldn’t even have doctor’s appointments, I couldn’t have hospital appointments which made me worry even more, and my husband’s a key worker so I was just on my own all the time. Yes, and I think that’s when it got to the point where I just felt like I couldn’t cope anymore.” – Mel*.

Several respondents recalled how this feeling of loneliness instilled a determination in them to retreat into isolation further. This meant they no longer wanted to speak to or be around others, even when they had a supportive network in place. An unstoppable cycle of isolation and socially avoidant behaviour was set in motion.

*“I just stopped talking to people. That’s when I stopped talking to anybody and I got really ill with my mental health because of it, but I thought “Well, why am I going to talk to people when they don’t listen to me anyway?”- Selina*.

*“I knew exactly what I was doing. I knew how I was going to do it. I just wanted it done. So I thought I have to tell him. I have to tell him. But I couldn’t tell him that I was off to kill myself.” – Simone*.

#### ‘Tired’ and ‘wired’

All but two respondents mentioned sleep deprivation as a major contributing factor to the accumulation of despair in the days or weeks before the suicide attempt. The sheer exhaustion they felt prevented them from thinking clearly or having the energy to face their circumstances and get better.

*“My little boy slept really well from, gosh, about three weeks, maybe less than that, he would sleep through the night which was really, really lucky, but I couldn’t sleep and I think, yes, the problems of not sleeping had a snowball effect.” – Hannah*.

This level of hypervigilance and restlessness was for many women the reason why they were unable to sleep. While women reported to feel exhausted on one hand, they also reported to experience an unhealthy level of drive, anger or arousal, leaving them ‘tired and wired’.

*“I stopped sleeping entirely; I was so angry all the time – it’s all the textbook depression symptoms, but I was so angry all the time. I was so tired all the time, but just wired, couldn’t sleep.”- Sam*.

*“I remember thinking I’m just so tired, I just want to go to sleep. I just want to be asleep and not be disturbed. But my mind was just so busy.” – Simone*.

Some displayed agitated and manic behaviour to such an extent that they struggled to understand how this went unnoticed.

*“I live three miles from the hospital and after they sent me home the next day, I walked back to the hospital with [the] kids and I was mowing the lawn five days after he was born and cleaning the house from top to bottom and driving all over the city after a [caesarean] section and you kind of just think like why did nobody notice? How can you think that that’s normal behaviour? Because I just felt this constant need, like I’ve got to be constantly doing things, constantly cleaning things, constantly walking places or doing things, alongside this absolute anger.” – Lauren*.

#### The irreversibility of motherhood

A majority of respondents described they came to a very agonising realisation that they were unable to get out of being a mother and that they found themselves in an irreversible situation, with no going back. The feeling of being ‘stuck’ was so pervasive, that many expressed they wanted either the pregnancy to end, or to not wake up. The irreversibility of motherhood was surrounded by feelings of deep regret and an admission that this had been their own fault and responsibility.

*“I remember actually hoping he would be stillborn towards the end, I think after the bridge. I just really wanted for him to be stillborn because if he was then it would all be over but it wouldn’t be my fault, and then I couldn’t go back. I think there was this constant sense of wanting to go back before any of it had happened and I just have my [older] children and I was working and I was happy and I kept seeking these ways just to go back and there weren’t any and I just got more and more desperate as time went on.” – Lauren*.

Many respondents shared their conflicting emotions towards their baby, who they viewed as the cause of their distress on the one hand, and as the reason to stay alive on the other.

*“[…] I simply could not do it anymore. Help, or don’t help. Whatever. I’m just not going to be around. And it’s almost like this feeling of, you want someone to take the baby off you, so that the baby’s not around, or that’s how I felt. The baby is your reason to stay alive, but the baby’s also the thing that’s causing you so much anguish. And that conflict is just so hard.” – Sam*.

Women were desperate to get a grip on the situation, yet it all felt in vain, with no improvement in sight. An overwhelming feeling of hopelessness took over, leaving women with no light at the end of the tunnel and only one option: taking their own life.

*“I don’t know how to explain it. I was feeling like all the things that I had to do were like water in my hands. I could see it. I could feel it. I could hold it. But it was coming through my fingers and I couldn’t do anything about it.” – Liv*.

## Discussion

Our study identified three overarching themes, marking different phases during which women’s mental health gradually deteriorated. Whilst not all sub-themes under these themes were necessarily reported by every respondent, they paint a comprehensive picture of the distressing feelings and contributing factors that women experienced in the days and weeks prior to their suicide attempt. Nearly half of our sample undertook a suicide attempt during pregnancy. This is in line with evidence suggesting antepartum suicide attempts are an important complication of pregnancy [2] and act as a strong predictor for postnatal suicidal behaviour, including completed suicide [41]. In addition, participants in our sample whose suicide attempt occurred during the postnatal period reported suicidal ideation had started during pregnancy, making the antenatal period a critical period for both antenatal and postnatal suicide prevention.

Our first theme, ‘Trauma and adversities’, captures vulnerabilities prior to conception and during pregnancy and has two key elements: (1) psycho-social adversities, including grief and trauma and (2) having a family history of perinatal mental health difficulties. Women with previous mental health difficulties, in particular those with a history of depression and mood disorders, are known to have an increased risk of fatal and non-fatal perinatal suicide attempts [3, 42, 43]. In addition, previous adverse life events and abuse, especially when these occurred during childhood, [44, 45], perinatal bereavement and infertility [46], comorbid substance use disorders and intimate partner violence [47], have also been associated with an increased risk of perinatal suicidal thoughts and suicidal behaviour. While the need for trauma-informed maternity services has become a public health priority [37], it is not always matched by a general awareness of the importance to raise these issues during pregnancy or the postnatal period [48]. This is reflected in our findings, where several of the respondents had experienced significant trauma and adverse life events prior to becoming pregnant but did not feel this was particularly relevant. Similarly, for some respondents a significant family history of perinatal mental health problems was unbeknown to them until their own mental health deteriorated. In contrast, those respondents who started pregnancy with an alertness of the risk of perinatal mental health problems in light of their own previous mental health difficulties or those of close relatives, reported to have prophylactic support measures in place, for instance by accessing a community perinatal mental health service during pregnancy. While this did not prevent their mental health from deteriorating, it did shorten the referral and escalation times when they reached a point of crisis. Having meaningful conversations about the prevalence of perinatal mental ill-health early on in pregnancy and undertaking a thorough assessment of mental health-related risk factors, such as previous mental health history, domestic abuse, substance misuse, previous trauma, among others, at every contact with maternity services is therefore essential to mitigate these pre-existing vulnerabilities [49].

In our second theme, ‘Disillusionment with Motherhood’, we identified a range of triggering factors that caused women’s mental health to decline. A first and often overlooked sub-theme that we identified was the impact of a physically and mentally challenging pregnancy and birth and their role in a subsequent mental health deterioration. This was often exacerbated when women received unkind, disrespectful care, which made them feel invisible. Whilst there are no studies to our knowledge that directly associate birth trauma with an increased risk of perinatal suicide, the association between birth trauma and postpartum post-traumatic stress disorder (PTSD) is well established [50–53]. Postpartum PTSD in turn is associated with poor coping and stress and highly co-morbid with depression [50]. Less evidence is available on the association between pregnancy and birth complications and perinatal suicide risk. One study found no association between maternal complications in pregnancy and during birth with hospitalisation for a suicide attempt [54]. Yet, as illustrated by our study sample, not all suicide attempts will result in an admission to a general hospital for medical treatment. Thus, further evidence is needed to understand the role of physical health complications, both during pregnancy, childbirth and the postnatal period, and their role in mood deterioration.

The subsequent sub-themes of ‘Invalidation of identify and self-sacrifice’ and ‘It wasn’t like starry-eyed love’ are closely intertwined and bring the complexity of women’s conflicting emotions towards motherhood to light [55]. The desire to be a good mother as a newly found identify often came to the detriment of their own personal self, with many women reporting situations of total self-sacrifice [56]. These daily struggles, of trying to be the perfect mother on the one hand, while trying to bond with their baby on the other hand, was in many cases fertile soil to start feeling obsolete as a person and feeling disillusioned in motherhood. Our findings build on previous work from Reid et al. (2022), who identified key factors in the context of a perinatal suicide attempt, such as a strained mother-infant bond, lack of social support, loneliness and hopelessness [44]. This resonates with our sub-themes of “Feeling like a failure”, “Alone in this world” and “Irreversibility of motherhood”. Our final theme “Entrapment and Despair” is in line with Reid et al. (2022)’s final phase, called ‘Darkness Descends’ [23] and is marked by pervasive feelings of hopelessness and failed motherhood. Under this theme, a turbulent accumulation of negative factors resulting in a fast deterioration of their mental health was reported by all respondents. These feelings of hopelessness and being totally entrapped were so all-encompassing, that participants felt no other way out than by attempting to take their own life. However, in this third stage, women did not just feel disillusioned, they felt totally incompetent as a mother, to a point they believed their baby and family would be better off without them. The finality of motherhood, with no way to turn back time or to escape their fate (‘Irreversibility of Motherhood’), drove them further to despair [55]. The MBRRACE-UK reports have repeatedly raised such feelings of incompetence as a mother and estrangement of the infant as a ‘red flag’ which should be taken seriously to prevent future maternal deaths by suicide [1, 13, 57].

Another factor we identified in this phase was the occurrence of intrusive thoughts and unsettling (psychotic) experiences, brought together in the subtheme ‘Intense intrusive thoughts and abnormal experiences’. The majority of our respondents reported abnormal experiences that were very unsettling to them. For some, these could be described as intrusive thoughts in the context of Obsessive Compulsive Disorder. Although intrusive thoughts are common among new parents, such experiences are often misunderstood, surrounded by stigma, and sometimes being misdiagnosed or over-normalised and dismissed, preventing timely and effective intervention [58]. For others in our sample, these experiences may have been delusions or hallucinations as part of a psychotic presentation. For all respondents who had them, the experiences were intense, frightening and difficult to understand at the time. Practitioner knowledge, sensitive risk assessment and careful diagnostic consideration about the nature and type of internal experiences is fundamental to appropriately treat women experiencing these upsetting experiences [59]. Yet equally important is increased public awareness on the occurrence and impact of such experiences, so women can seek timely support when they experience these frightening thoughts or delusions.

A third common factor we identified was sleep deprivation during pregnancy and the postnatal period and its profound impact on women’s mental health. Sleep disturbance is very common in relation to mental illness, and was highlighted in the most recent MBRRACE-UK report as marked and persistent in those women who died by suicide, even when treated with hypnotic medication [1]. A recent systematic review by Palagini et al. (2023) showed insomnia and poor sleep quality increased the odds of suicidal risk in pregnant and postpartum women by more than threefold, independently from psychiatric comorbidity [60]. Especially in a context of onset of psychotic illness, such as bipolar disorder, insomnia often precipitates other psychotic symptoms such as restlessness, irritability and rapid mood changes [61]. Unfortunately, as sleep loss is generally accepted as a common ‘side effect’ of pregnancy and having a newborn baby, its severity and potential devastating consequences are poorly understood and often minimised. Overall, the theme of ‘Entrapment and Despair’ captures the sheer hopelessness and inability to gain control over a rapidly escalating situation, in line with Klonsky and May’s Three-Step-Theory of suicide [62]. This theoretical model of suicide considers three steps to suicide. Being in pain and hopelessness leads to suicidal ideation (Step 1), which can be exacerbated by isolation or countered by connectedness (Step 2). The final step is marked by one’s capability of attempting suicide (Step 3). The pervasive feeling of hopelessness and lack of control gradually paved the way for a solid belief it would be better to no longer be here. Participants in our sample shared how they accepted this belief and waited for an opportunity to carry out their suicide plan. This combination of hopelessness and rejection of motherhood, a belief that death would be preferred and an opportunity to act on these thoughts has been previously theorised as a culmination of factors for perinatal suicide [23]. In line with findings from previous MBRRACE-UK reports, the vast majority of respondents in our sample turned to violent methods for suicide, such as jumping, hanging, suffocation, using sharp objects or stepping in front of traffic, reflecting the high level of distress women found themselves in and the determination with which they wanted to carry out their plan.

### Strengths and limitation

Our study is the first to our knowledge to focus on suicide attempts during the perinatal period and offers a rich understanding of women’s experiences surrounding these highly distressing events. A strength of this study is the recruitment of participants across the UK, rather than one geographical area, with diversity in the sample regarding age, parity, psychiatric morbidity, social support, educational attainment and socio-economic status. Significant efforts were made to recruit women from diverse ethnic, cultural, and religious backgrounds, through invitations and meetings with community leaders and designated support groups. Despite our efforts, we did not achieve diversity regarding ethnic and cultural background, one of the limitations of this study. Ethnicity data from the latest MBRRCACE-UK report showed that women who died by suicide were predominately white (86%), with no further ethnicity details on the remaining 14% [1]. As a result, although this was never our intention, we are aware our study findings are focusing on the experiences of White women in the UK and not transferrable to an ethnically and culturally more diverse sample, or to other countries across the globe. In addition, our sample consisted predominantly of participants with higher educational qualification, in positions of employment. Therefore, our analysis was unable to explore the impact of poverty on women’s suicidality, which is known to be an important driver of poor (perinatal) mental health [63, 64]. We are aware a more ethnically, culturally, religiously and socio-economically heterogenous sample is likely to represent a diversity of perspectives, highlighting these issues. Another limitation was the design of the demographic survey, which did not specifically differentiate between mental health diagnosis given during the life course or specifically at the time of the suicide attempt. Suicide research, especially in a perinatal context, is notorious for its recruitment challenges. Saturation for all themes and sub-themes was achieved well within the available sample and is one of the strengths of this study. Another strength of our study is the sensitivity and rigour of patient and public involvement throughout the various phases of the study. This was crucial to do justice to the courage which participants had shown by sharing their stories and to keep respondents safe throughout their research participation.

### Implications for clinical practice and care and future research

Our study highlights the importance of routine inquiry of previous mental health difficulties and family history of perinatal mental health problems at the first encounter during pregnancy. Yet, such an assessment needs to be more comprehensive than a tick-box exercise and should be accompanied by a personalised conversation about prevalence of perinatal mental health problems and potential triggers, including trauma and grief. Professionals should be given adequate time during antenatal encounters to explore this in depth and, where required, receive additional training in perinatal mental health to build confidence in doing so. The perinatal period is often described as a ‘window of opportunity’, but this goes both ways: While every encounter creates opportunity for screening, detection and support, it also has the potential for invoking or deepening trauma. Our study revealed the devastating and long-lasting impact of unkind, careless and dismissive remarks by healthcare professionals on women’s mental health, thus instilling a feeling of failure by throw-away comments that would ripple on weeks and months after they were uttered. Perinatal healthcare professionals need to understand the weight of their words, how they can provide hope when women are struggling, but equally how they can push women further into isolation and despair. Culturally aware and trauma-informed clinical practice is essential to achieve this, whilst also recognising the impact of burn-out and carer’s fatigue in an overstretched and under-resourced healthcare service. Healthcare professionals need to be cautious about the difference between *normalising* and *dismissing* distressing feelings. In addition, professionals need to fully understand the profound impact of physical, social and psychological risk factors as identified by our study. The physical and mental challenge of pregnancy and childbirth, often in combination with a traumatic birth experience should not be underestimated. An impaired mother-infant dyad, feelings of resentment of motherhood, and the discrepancy between women’s expectations and their lived reality are all key triggers that should be discussed, identified and addressed at the earliest opportunity, in a non-judgemental and sensitive way to avoid further escalation. Women need to be validated and reassured by professionals when disclosing these feelings, and be informed that support is available to help them transition into motherhood. Continuity of care throughout the perinatal period, if done with sensitivity and person-centredness, can foster trusting relationship so women feel safe and supported to disclose distressing feelings. Similarly, insomnia and sleep disturbance, albeit in combination with restlessness and irritability, intrusive thoughts and feelings of lack of control and failure are red flags for severe and rapid mental health deterioration that required prompt and effective action. More than anything, women need to feel safe and listened to, so they can share their feelings with healthcare professionals without fear of judgement, shame and stigma. Our study showed that women will often retreat into silence prior to a suicide attempt and in that moment more than ever rely on attentive, educated and compassionate support networks to avoid a suicide attempt.

Future research into perinatal suicide attempt should focus on developing effective preventative interventions and public health strategies, both in an antenatal and postnatal context, with their distinct healthcare professionals’ involvement and resource challenges. By using implementation science methods, these interventions should be tested and evaluated on their efficacy and effectiveness, in order to reduce future maternal suicides.

## Conclusion

This study is the first UK-based qualitative study looking at suicide attempts during the perinatal period. Our findings identified three themes with several contributing factors which led women to undertake a suicide attempt. It is important to understand the impact of previous trauma and life adversity when going through pregnancy and the postnatal period. Feelings of disillusionment with motherhood and feeling entrapped in a hopeless situation were key phases women experienced in the lead-up to their suicide attempt. Our study findings have important implications for clinical practice and healthcare professionals should be aware of warning signs, to improve timely detection and facilitate meaningful inquiry, in order to improve care and prevent future maternal suicide deaths.

### Electronic supplementary material

Below is the link to the electronic supplementary material.

Supplementary Material 1

## Data Availability

The datasets generated and analysed during the current study are not publicly available due to the privacy of the participants in the study and the sensitive nature of the data. Further inquiries can be directed to the corresponding author (abigail.easter@kcl.ac.uk).

## Footnotes

1 We acknowledge not all people who give birth and go through the perinatal period identity as women, female or mothers. While this paper utilises predominantly the terms women and mothers, we aim to include also those who identify as transgender, non-binary or any other gender identity.
