# Supplementary material for: Women’s experiences of attempted suicide in the perinatal period (ASPEN-study) – a qualitative study
Source: BMC Psychiatry. 2024 Apr 3;24:255. doi: 10.1186/s12888-024-05686-3 (PMC10988966; doi:10.1186/s12888-024-05686-3)
Supplement: Supplementary file 1 — Supplementary Material 1 [file 12888_2024_5686_MOESM1_ESM.docx]

# ANNEX I Table of supplementary quotations

| **Phase 1: Trauma and Adversities** | **Phase 2: Disillusionment with Motherhood** | **Phase 3: Entrapment and Despair** |
| --- | --- | --- |
| **Psycho-social adversities**  “I’d had suicidal thoughts before; I’ve gone and stood on a bridge when I’ve been very depressed, multiple times [laughs] historically, that people knew about, it’s in my records and stuff.” – Sarah  So I’ve had depression in the past, but it was a long time ago, so sort of late teens, early 20s I really struggled with depression for several years and I actually had a suicide attempt I think when I was about 21 and I’ve spent a long time on antidepressants, different antidepressants, which I think now aren’t prescribed, like Prozac, and I was under psychiatry services and I felt as though I’d done a lot of work to, I guess, learn about myself, learn about different coping mechanisms, I’d had CBT in the past, I’d had counselling. I feel as though I’ve been on a bit of a lifelong journey in terms of my self-development. – Rosy  “So a little bit of context. Before that pregnancy I had another kid and it took me a while to adjust to motherhood, everything, all the changes and everything. And one friend that was diagnosed with postpartum depression after she had her kid, she suggested maybe that’s also what happened to me.” – Liv  **Family history of perinatal mental illness**  “My younger sister, I’ve got two sisters, two were… Three were struggling with mental health, two had addiction (me and my sister) – problems, and both tried committing suicide during pregnancy.” – Anna | **Physical and mental challenge of pregnancy and birth**  “I was huge as well, I put a lot of weight on, I had a lot of water retention so I was very big, my belly was very heavy, and they would have us going here, there and everywhere. It was a proper struggle.” – Selina  “I have horrible pregnancies. I have HG normally. What’s the name – like when your hips open too much and you have pain, PGP? That’s normal, my normal pregnancies, I´m quite petite and the pregnancy puts a lot of pressure on my body.” – Liv  “So I’d been induced and they gave me the first lot of pessaries and it was like from naught to full labour in literally minutes. So there was no adjustment. It was just like, my God, this is the most horrendous thing ever.” – Simone  **Invalidation of Identify and Self-Sacrifice**  “I felt as though I was being grilled. I was trying to say, “I’m struggling, I need some help,” but I felt as though she wasn’t hearing me. It was all about, “Have you fed this baby? Is it…?” And I’m like, “Yeah, the baby’s being fed, it’s on formula now. He’s eating, he’s sleeping, he’s putting weight on,” and she was just mithering, mithering, mithering about, “Right, well you need to go to this group at the children’s centre”. And I’m thinking ‘I can’t even get a brush through my hair. I feel as though I’m behind in what I’m supposed to be doing. I’ve only just managed to get to grips with feeding, nappy changing, getting the baby dressed. It’s sat in its car seat when I’m in the shower. It’s all a bit… I haven’t got time and I can’t think about that. But she was pushing it and pushing it. And I said, “I can’t. I just can’t”. And she made this comment where she said, “You need to engage in this, [respondent], or you’ll find that your children will be placed in special measures”.” – Simone  “I was really trying to be something that wasn’t sustainable and I think a lot of women do do that in the beginning, don’t they, when they have a newborn, they try and be this perfect mum. But what is perfect?” – Hannah  “I kept just feeling like I should have done something, I should have been able to get her out safely, I should have been able to deliver her, I should have done something, I should have told them I wanted… All these crazy thoughts were going through my mind, and that’s when I started to feel really sad.” – Mel  **‘It was not like starry-eyed love’**  “And it didn’t really come very naturally to me; it’s a very foreign, alien concept, that there’s this human growing inside of you. But we found out the gender, we picked a name, and tried to do all the bonding things. But it was really hard. It was really hard to feel anything close to excitement about having the baby.” – Sam  “In terms of motherhood, yes, [pause] I don’t know whether I just felt I was failing at it or… [pause] I don’t know, I felt very [pause] not connected to the baby. I had felt very, very bonded and very connected, and then I wasn’t at all.” – Sarah | **Feeling like a failure**  “I felt like a real failure, like “You couldn’t give birth to her, and you can’t even feed her; what kind of mum are you?” I remember that going through my mind quite a lot.” – Mel  “So there were many times that I thought, if I just died now, nobody would have to…I wouldn’t be feeling as crap as I was feeling. And seeing my husband trying to juggle everything. Okay that is one thing that he wouldn’t have to worry about.” – Liv  **Intense intrusive ideas and abnormal experiences**  “But it just all escalated so quickly, and I was hearing things, I was hallucinating, yes. You just feel unhinged. The way that it’s portrayed in movies and stuff; totally erratic, totally unhinged, there was no rational thinking whatsoever. Just like an out of body experience.” – Sam  “Nobody had mentioned intrusive thoughts. If somebody had mentioned at all the words ‘intrusive thoughts’ [laughs] or explained what they were, particularly with someone who’s sat down with a Perinatal Psychiatrist saying [laughs] “I’m worried about…” and I wasn’t even familiar with this, so they clocked that I might get postnatal something and that they were going to give me antipsychotics, but nobody sat down and said “These are all the different ways that could manifest, these are all the different things. If this happens, you’re not on your own and it’s common,” and I don’t know if I’d known that, then…” – Sarah  **Alone in this world**  “My husband started a new job, so he was on nights pretty much as soon as we went home, so I was having to sort of do a lot myself anyway.” – Hannah  “I was probably spending too much time on my own, so could have done with some company. If someone had been in the house I wouldn’t have done it.” – Marie  “I look back now and I think why didn’t you just tell someone, why didn’t you kind of just talk to someone about it? And I don’t know what it was with me at the time, I don’t know whether I didn’t recognise it or I was ashamed of it, but I just didn’t feel able to talk to anybody at all.” – Lauren  **‘Tired’ and ‘wired’**  “I think a lot has to do with the lack of sleep. Like, because I was feeling unwell in general, my back, my hips, my ribs, everywhere was sore. It was really hard for me to get a good night’s sleep so I definitely think that that would have something to do with it.” – Liv  “I couldn’t sleep because [youngest daughter] was crying all the time and wouldn’t be laid down, but then when she did start to maybe rest or have periods when she was resting I just couldn’t sleep, and that went on for months and I think getting so, so sleep deprived, that anxiety, the whole thing just resulted in me really being really poorly but not letting people know how poorly I was.” – Rosy  “So if I did nod off in the night, I’d just wake up with a jump and have to prod her just to make sure; it was really intense, so I’d stay awake, because she was next to me in the cot thing next to the bed and yes, she was such a good sleeper that I kept thinking “She’s not asleep.” That was my biggest fear, I think, that if I fell asleep and I wasn’t watching her, that’s when it would happen. And yes, it was just exhausting, it was absolutely exhausting. And I wasn’t sleeping, I wasn’t really eating, I wasn’t going out much.” – Mel  **The irreversibility of motherhood**  “[…] it was just like I just did not want to be, I did not want to be a mum, I did not want to be there, I did not want to be responsible. That’s what it was, I didn’t want to be responsible.” – Sarah  “Something clicked in my head that was like you can’t get out of this, you can’t manage your way out of this, you are now a mum to this baby. So there’s no giving it back, there’s no changing your mind, there’s no this was a really bad decision, let’s try something else. It’s like no, you have to crack on.” – Simone  It was this doom. It was this absolute – I used to describe it as like a black cloud, that just hung over me all the time. And every single day, I would wake up, and I would think, “Oh, shit, I’m a day closer to having this baby.” And it caused all those feelings of regret. But you can’t ever say those things out loud, because people will assume that you want an abortion, or that you made a reckless decision, or – you know, you wanted this. It’s very much like, “You chose this; you wanted this; this is your lot.” And it got really bad. It got to the point where, prenatally, I would just genuinely hope that I wouldn’t wake up the next day. Because that was a day closer to having the baby.” – Sam  “I think the overriding thought was that I’d brought this situation on myself, so I needed to get on with it and control it.” – Rosy |
